# The Proinflammatory and Proangiogenic Macrophage Migration Inhibitory Factor Is a Potential Regulator in Proliferative Diabetic Retinopathy

**DOI:** 10.3389/fimmu.2019.02752

**Published:** 2019-12-04

**Authors:** Ahmed M. Abu El-Asrar, Ajmal Ahmad, Mohammad Mairaj Siddiquei, Alexandra De Zutter, Eef Allegaert, Priscilla W. Gikandi, Gert De Hertogh, Jo Van Damme, Ghislain Opdenakker, Sofie Struyf

**Affiliations:** ^1^Department of Ophthalmology, College of Medicine, King Saud University, Riyadh, Saudi Arabia; ^2^Dr. Nasser Al-Rashid Research Chair in Ophthalmology, College of Medicine, King Saud University, Riyadh, Saudi Arabia; ^3^Department of Microbiology and Immunology, Rega Institute for Medical Research, KU Leuven, Leuven, Belgium; ^4^Laboratory of Histochemistry and Cytochemistry, KU Leuven, Leuven, Belgium

**Keywords:** proliferative diabetic retinopathy, migration inhibitory factor, CD74, angiogenesis, Müller cells

## Abstract

The macrophage migration inhibitory factor (MIF)/CD74 signaling pathway is strongly implicated in inflammation and angiogenesis. We investigated the expression of MIF and its receptor CD74 in proliferative diabetic retinopathy (PDR) to reveal a possible role of this pathway in the pathogenesis of PDR. Levels of MIF, soluble (s)CD74, soluble intercellular adhesion molecule-1 (sICAM-1) and vascular endothelial growth factor (VEGF) were significantly increased in the vitreous from patients with PDR compared to nondiabetic control samples. We detected significant positive correlations between the levels of MIF and the levels of sICAM-1 (*r* = 0.43; *p* = 0.001) and VEGF (*r* = 0.7; *p* < 0.001). Through immunohistochemical analysis of PDR epiretinal membranes, significant positive correlations were also found between microvessel density (CD31 expression) and the numbers of blood vessels expressing MIF (*r* = 0.56; *p* = 0.045) and stromal cells expressing MIF (*r* = 0.79; *p* = 0.001) and CD74 (*r* = 0.59; *p* = 0.045). Similar to VEGF, MIF was induced in Müller cells cultured under hypoxic conditions and MIF induced phosphorylation of ERK1/2 and VEGF production in Müller cells. Intravitreal administration of MIF in normal rats induced increased retinal vascular permeability and significant upregulation of phospho-ERK1/2, NF-κB, ICAM-1 and vascular cell adhesion molecule-1 expression in the retina. MIF induced migration and proliferation of human retinal microvascular endothelial cells. These results suggest that MIF/CD74 signaling is involved in PDR angiogenesis.

## Introduction

Ischemia-induced retinal angiogenesis and excessive deposition of extracellular matrix lead to the formation of fibrovascular membranes at the vitreoretinal interface in proliferative diabetic retinopathy (PDR). This outgrowth of fibrovascular tissue, composed of new blood vessels, leukocytes and α-smooth muscle actin (α-SMA)-expressing myofibroblasts (1–4), often causes serious vision loss due to recurrent vitreous hemorrhage and/or traction retinal detachment. Several studies support the paradigm that inflammation, neovascularization and fibrosis are critical mechanisms for PDR initiation and progression as the authors showed overexpression of inflammatory, angiogenic and fibrogenic factors inducing those processes (1–5).

In PDR, hypoxia seems to drive neovascularization through upregulation of angiogenic factors (6, 7). In particular vascular endothelial growth factor (VEGF), upregulated in retinal cells in response to oxygen deprivation (8, 9), plays a pivotal role in promoting retinal neovascularization and vascular leakage (10, 11). In addition to angiogenesis, recruitment of leukocytes occurs in the ocular microenvironment of patients with PDR (1–5). Recent data support a causal relationship between persistent inflammation and angiogenesis (12, 13) and this interplay might also be critical for PDR development and progression. Accordingly, some of the signaling molecules of the inflammatory response, such as cytokines, chemokines and their receptors might play an essential role in PDR angiogenesis and progression. Therefore, a new challenge in PDR research is the identification of the molecular links between inflammation and angiogenesis.

Macrophage migration inhibitory factor (MIF) is a widely expressed proinflammatory cytokine originally discovered as a product isolated from the culture medium of activated T lymphocytes that inhibited the random migration of cultured macrophages *in vitro* (14). Today a wide spectrum of biological properties has been attributed to MIF. MIF is closely involved in autoimmune and inflammatory diseases (15–17). The biological effects of MIF are mediated through its primary receptor CD74, which is the major histocompatibility class II-associated invariant chain (18). The binding of MIF to its receptor CD74 leads to the activation of extracellular signal regulated kinase (ERK) 1 and 2 and the proinflammatory transcription factor nuclear factor-κB (NF-κB) (15). Recently, it was demonstrated that the MIF/CD74 signaling pathway promotes macrophage-mediated inflammation in type 1 diabetes (19). In addition, the chemokine receptors CXCR2 and CXCR4 were identified as functional receptors for MIF. By activating CXCR2 and CXCR4, MIF displays chemokine-like functions and stimulates leukocyte chemotaxis (20).

Besides its role in inflammation, MIF has potent proangiogenic properties and in conjunction with its cell surface receptor CD74 emerges as an important regulator of pathological angiogenesis associated with several types of malignant tumors (21–23). Exogenous MIF stimulated *in vitro* endothelial cell migration, proliferation and tube formation, key steps in the angiogenesis cascade (24–26). Furthermore, MIF induced angiogenesis in multiple *in vivo* models (25). Several studies reported overexpression of MIF and CD74 in multiple cancers (21–23, 27) and that the MIF/CD74 signaling pathway promotes tumor progression and angiogenesis (21–24, 27–33). Furthermore, anti-MIF antibodies suppressed angiogenesis in animal models of cancer (33). Recently, it was demonstrated that small molecule inhibitors of MIF, inhibit cancer development in animal models (29, 34), reduce the severity of experimental autoimmune encephalomyelitis (17) and lower blood glucose in an animal model of non-insulin-dependent diabetes mellitus (35). MIF was proposed as the link that connects the inflammatory response to tumor-associated angiogenesis (24, 28–31). The angiogenic activity in tumors was attributed to the fact that MIF acts as a potent inducer of the angiogenic factors VEGF, CXCL5, and CXCL8 in tumor cells (21, 28, 30).

Given the key roles of the MIF/CD74 signaling pathway in angiogenesis and inflammation, we hypothesized that this pathway may be involved in the pathogenesis of PDR. To test this hypothesis, we investigated the expression of MIF and CD74 in the ocular microenvironment of patients with PDR and correlated their levels with the angiogenic activity in epiretinal fibrovascular membranes and the vitreous levels of VEGF and the inflammatory biomarker soluble intercellular adhesion molecule 1 (sICAM-1). We examined the effect of intravitreal administration of MIF on the retinas from normal rats. We analyzed *in vitro* the expression of MIF in human retinal Müller glial cells following exposure to hydrogen peroxide (H_2_O_2_)-induced oxidative stress and the hypoxia mimetic agent cobalt chloride (CoCl_2_) and monitored expression of VEGF in Müller cells following exposure to MIF. Finally, we investigated the response of human retinal microvascular endothelial cells (HRMECs) to treatment with MIF.

## Materials and Methods

### Patient Samples

Undiluted vitreous fluid samples were obtained from 36 patients with PDR during pars plana vitrectomy for the treatment of tractional retinal detachment, and/or nonclearing vitreous hemorrhage and processed as described previously (1–5). The diabetic patients were 23 males and 13 females whose ages ranged from 27 to 74 years with a median [IQR] of 54 [44-59]. The PDR group consisted of 20 patients who had insulin-dependent diabetes mellitus and 16 patients who had non-insulin-dependent diabetes mellitus. Vitreous fluid samples obtained from 20 patients who had undergone vitrectomy for the treatment of rhegmatogenous retinal detachment with no proliferative vitreoretinopathy (PVR) were used as the control samples. Clinical check-up confirmed that control subjects were free from diabetes or other systemic disease. The controls were 14 males and 6 females whose ages ranged from 26 to 73 years with a median [IQR] of 55 [38-66]. The ages (*p* = 0.526; Mann-Whitney test) and male/female ratios (*p* = 0.547; Chi-Square test) did not differ significantly between nondiabetic control patients and PDR patients.

Fourteen patients with PDR undergoing pars plana vitrectomy for the repair of tractional retinal detachment donated epiretinal fibrovascular membranes. At the time of the procedure, using previously published criteria, retinal neovascular activity was clinically graded (36). We made a distinction between active neovascularization (visible perfused new vessels on the retina or optic disc present within epiretinal membranes) and inactive involuted disease (nonvascularized, white fibrotic epiretinal membranes). For comparison, epiretinal fibrocellular membranes were obtained from ten patients without diabetes undergoing vitreoretinal surgery for the treatment of retinal detachment complicated by PVR. The epiretinal membranes were processed as previously described (1–4).

The study was conducted according to the tenets of the Declaration of Helsinki. Before undergoing vitrectomy, all patients signed a preoperative informed written consent and approved the use of the excised epiretinal membranes and aspirated vitreous fluid for further analysis and clinical research. The Research Center and Institutional Review Board of the College of Medicine, King Saud University approved the study design and protocol.

### Immunohistochemical Staining of Human Epiretinal Membranes and Quantitations

For CD31, α-SMA and MIF detection, antigen retrieval was performed by boiling the sections in citrate based buffer [pH 5.9–6.1] [BOND Epitope Retrieval Solution 1; Leica, Diegem, Belgium] for 10 min. For CD45 and CD74 detection, antigen retrieval was performed by boiling the sections in Tris/EDTA buffer [pH 9] [BOND Epitope Retrieval Solution 2; Leica] for 20 min. Subsequently, the sections were incubated for 1 h with mouse monoclonal anti-CD31 (ready-to-use; clone JC70A; Dako, Glostrup, Denmark), mouse monoclonal anti-CD45 (ready-to-use; clones 2B11+PD7/26; Dako), mouse monoclonal antibody against α-SMA (ready-to-use; clone 1A4; Dako), mouse monoclonal anti-CD74 antibody (1:50; ab9514, Abcam, Cambridge, UK) and rabbit polyclonal anti-MIF antibody (1:200; ab65869, Abcam). Optimal working conditions for the antibodies were determined in pilot experiments on kidney, tonsil and liver sections. The sections were then incubated for 20 min with an alkaline phosphatase-conjugated IgG. Immune interactions were visualized with the Fast Red chromogen (Leica; 15 min incubation). Finally, a faint counterstain with Mayer's hematoxylin was performed.

To identify the phenotype of cells expressing MIF and CD74, sequential double immunohistochemistry was performed. The sections were first incubated with anti-CD45, followed by treatment with peroxidase-conjugated secondary antibody and 3, 3′-diaminobenzidine tetrahydrochloride substrate. Next, the second primary antibodies (anti-MIF or anti-CD74) were added and detected by alkaline phosphatase-conjugated secondary antibody and Fast Red reactions. No counterstain was applied. In negative controls, the incubation step with primary antibody was omitted from the protocol and only the ready-to-use antibody diluent (Cat No 52022; Dako) was applied.

Immunoreactive blood vessels and cells were counted in five representative fields, using an eyepiece calibrated grid in combination with the 40x objective as previously described (1–4). The level of vascularization in epiretinal membranes was determined by immunodetection of the vascular endothelium marker CD31.

### Enzyme-Linked Immunosorbent Assays

Enzyme-linked Immunosorbent Assay (ELISA) kits for human MIF (Cat No DMF00B), human VEGF (Cat No SVE00) and human sICAM-1 (Cat No SCD540) were purchased from R&D Systems (Minneapolis, MN, USA). Levels of human MIF, VEGF and sICAM-1 in vitreous fluid and MIF and VEGF in culture medium were determined using those ELISA kits according to the manufacturer's instructions. The minimum detection limits for MIF, VEGF and sICAM-1 ELISA kits were 0.016 ng/ml, 9 pg/ml and 0.096 ng/ml, respectively.

### Human Retinal Müller Glial Cell and Retinal Microvascular Endothelial Culture

Human retinal Müller glial cells (MIO-M1) (a generous gift from Prof. A. Limb, Institute of Ophthalmology, University College London, UK) were cultured with DMEM containing 1 g/L glucose with 10% (v/v) fetal bovine serum and 1% penicillin/streptomycin. Confluent cells were starved overnight in serum-free DMEM to minimize the effects of serum and subsequently either left untreated or stimulated for 24 h. The following stimuli were used: recombinant human MIF (2, 20 or 100 ng/ml) (Cat No: 289-MF, R&D Systems), 100 μM H_2_O_2_ (Cat No: H101351000, Scharlau, Sentmenat, Spain) or 300 μM CoCl_2_ (AVONCHEM Limited, Nacclesfield, Cheshire, UK). Human retinal microvascular endothelial cells (HRMECs; Cell Systems, Kirkland, WA, USA) were cultured in Endothelial Cell Basal Medium-2 (EBM-2) supplemented with the EGM-2 MV SingleQuots kit (both from Lonza, Verviers, Belgium).

### Intravitreal Injection of MIF

Intravitreal injection into the eyes of Sprague Dawley rats (220–250 g) was performed as previously described (37). While the animals were kept under deep anesthesia, 5 μl of sterilized solution containing 5 ng recombinant MIF or sterile phosphate buffer saline (PBS) was injected into the right or left eye, respectively. Four days after intravitreal administration the rats were sacrificed, retinas were carefully dissected, snap frozen in liquid nitrogen, and stored at −80°C.

### Measurement of Blood-Retinal Barrier Breakdown

Blood-retinal barrier (BRB) breakdown in excised retinas was evaluated 4 days after intravitreal injection as previously described (37). Briefly, deeply anesthetized rats were intravenously injected with 50 mg/kg fluorescein isothiocyanate (FITC)-conjugated dextran (3–5 kDa, Sigma-Aldrich Corp., St. Louis, MO, USA). After 30 min, a blood sample was collected, and each rat was then perfused with PBS. The retinas were carefully excised, weighed and homogenized to extract the FITC-conjugated dextran. Fluorescence was measured using a spectra Max Gemini-XPS microplate reader (Molecular Devices, Sunnyvale, CA, USA) with excitation and emission wavelengths of 485 and 538 nm, respectively, with PBS as a blank. A correction for autofluorescence was made by subtracting the autofluorescence of retinal tissue from non-treated rats. The concentration of FITC-conjugated dextran in each retina was calculated from a standard curve of FITC-conjugated dextran in water. For normalization, the retinal FITC-conjugated dextran amount was divided by the retinal weight and by the concentration of FITC-conjugated dextran in the plasma. BRB breakdown was calculated using the following equation, with the results being expressed in μl/(g*h).

$$\begin{array}{l} \frac{Retinal\;FITC-dextran\;\left(\mu g\right)/retinal\;weight\;\left(g\right)}{Plasma\;FITC-dextran\;concentration\;\left(\mu g/\mu l\right){\;}^{*}circulation\;time\;\left(h\right)} \end{array}$$

### Western Blot Analysis of Human Vitreous Fluid, Müller Cell Lysates and Rat Retinas

Retina and cell lysates were homogenized in western blot lysis buffer [30 mM Tris-HCl; pH 7.5, 5 mM EDTA, 1% Triton X-100, 250 mM sucrose, 1 mM Sodium vanadate, and a complete protease inhibitor cocktail from Roche (Mannheim, Germany)]. After centrifugation of the homogenates (14,000 × g; 15 min, 4°C), protein concentrations were measured in the supernatants (DC protein assay kit; Bio-Rad Laboratories, Hercules, CA). Equal amounts (50 μg) of the protein extracts were subjected to SDS–PAGE and transferred onto nitrocellulose membranes. To determine the presence of soluble (s) CD74 in the vitreous samples, equal volumes (15 μl) of vitreous samples were boiled in Laemmli's sample buffer (1:1, v/v) under reducing conditions for 10 min and analyzed as described (2–4).

Immunodetection was performed with the use of mouse monoclonal anti-CD74 antibody (1:1,000; ab-9514, Abcam), rabbit monoclonal anti-phospho-ERK1/2 antibody (1:1,500; MAB1018; R&D Systems), mouse monoclonal anti-p65 subunit of NF-κB antibody (1:500, sc-136548, Santa Cruz Biotechnology Inc, Santa Cruz, CA, USA), mouse monoclonal anti-ICAM-1 antibody (1:500, sc-8439, Santa Cruz Biotechnology Inc), mouse monoclonal anti-vascular cell adhesion molecule-1antibody (VCAM-1) (1:500, sc-13160, Santa Cruz Biotechnology Inc) and mouse monoclonal anti-VEGF antibody (1:750, MAB293, R&D Systems). Nonspecific binding sites were blocked (1.5 h, room temperature) with 5% non-fat milk made in Tris-buffered saline containing 0.1% Tween-20 (TBS-T). Three TBS-T washings (5 min each) were performed before the secondary antibody treatment at room temperature for 1 h. Immune reactive bands were visualized with luminol reagent (sc-2048; Santa Cruz Biotechnology Inc.). To verify equal loading, membranes were stripped and reprobed with β-actin-specific antibody (1:2,000; sc-47778; Santa Cruz Biotechnology Inc.). Band intensities were quantified using GeneTools software (Syngene by Synoptic Ltd., Cambridge, UK).

### Chemotaxis Assay

Chemotaxis of HRMECs was evaluated with an xCELLigence system (ACEA Biosciences, San Diego, CA) as described before (38). Migration was evaluated in response to different concentrations of recombinant human MIF (Cat No 289-MF, R&D Systems), 10 ng/ml of VEGF (Cat No 583702, Biolegend, San Diego, CA) or dilution medium. First, 160 μl of stimulus diluted in MCDB131 medium (Gibco/ThermoFisher Scientific, Waltham, MA, USA) supplemented with 0.4% (v/v) fetal calf serum were added to the wells of the lower chamber of a Cell Invasion/Migration (CIM)-Plate (ACEA Biosciences). After assembly of the lower and upper chamber, 50 μl of serum-free MCDB131 medium was added in the upper wells. After equilibration of the plate (1 h at 37°C), HRMECs were added in the upper chamber at 4 × 10^4^ cells in 100 μl/well. After an additional incubation period (30 min at room temperature) to allow settling of the cells, migration was monitored every minute for 15 h in the xCELLigence apparatus. Cell migration from the upper to the lower compartment was recorded as changes in electrical impedance. These changes were converted into cell indices, as a measure of cell migration.

### Proliferation Assay

To assess the proliferative effect of MIF, HRMECs were seeded at 5 × 10^3^ cells in 100 μl/well of a 96-well plate in culture medium (*vide supra*). The next day, cells were washed with serum-free MCDB131 medium and stimulated in proliferation medium [MCDB131 medium supplemented with 2mM GlutaMAX^TM^ (Gibco), 30 μg/ml Gentamicin and 3% fetal calf serum]. Either different concentrations of MIF, 10 ng/ml VEGF or proliferation medium were added to the wells. After 48 h, cell proliferation was assessed using the ATPlite Luminescence Assay kit (PerkinElmer, Waltham, MA) according to the manufacturer's instructions.

### Statistical Analysis

Statistical analyses of the data were performed using SPSS version 21.0. Normal distribution of the data was verified using the Shapiro-Wilk (S-W) test and normal Q-Q plots. Normally distributed data were presented as mean ± SD and an independent *t*-test was used to compare the groups. For normally distributed data, Pearson correlation coefficients were calculated. Non-parametric tests (Kruskal-Wallis test, Mann-Whitney test and Spearman's correlation coefficients) were performed for not normally distributed data, which were presented as median and interquartile range [IQR; Q1–Q3]. Proportions were compared using the Chi-Square test. The level of statistical significance was set at 0.05.

## Results

### Analysis of Angiogenic and Inflammatory Activities and the Expression of the Myofibroblast Marker α-SMA in Epiretinal Fibrovascular Membranes From Patients With PDR

**Figure 1:**
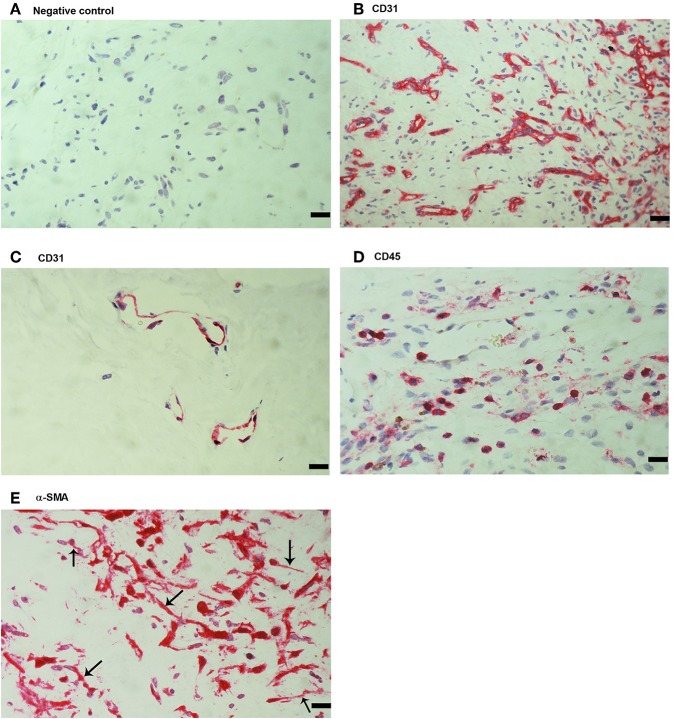
Detection of endothelial cells, leukocytes and myofibroblasts in proliferative diabetic retinopathy (PDR) epiretinal fibrovascular membranes. No staining was observed in the negative control slide following the full staining procedure with omission of the primary antibody from the staining protocol **(A)**. Immunohistochemical staining for CD31 showing pathologic new blood vessels expressing this endothelial cell marker in a membrane from a patient with active neovascularization **(B)** and in a membrane from a patient with involuted PDR **(C)**. Note that the membrane from the patient with involuted PDR is composed mostly of fibrous tissue. Immunohistochemical staining for the leukocyte common antigen CD45 showing numerous leukocytes in the stroma **(D)**. Immunohistochemical staining for α-smooth muscle actin (α-SMA) showing immunoreactivity in myofibroblasts (arrows) **(E)**. (Scale bar, 10 μm).

As a negative control, the experimental staining procedure was performed with omission of the primary antibody and no staining was observed (Figure 1A). Subsequently, we used staining for the vascular endothelial cell marker CD31, the leukocyte common antigen CD45 and α-SMA to evaluate ongoing angiogenesis, inflammation, and fibrosis, respectively. All membranes showed neovessels positive for CD31 (Figures 1B,C). Representative examples of CD31 staining in membranes with active (Figure 1B) and involuted (Figure 1C) disease are shown. Furthermore, leukocytes expressing CD45 (Figure 1D), as well as spindle-shaped cells expressing α-SMA (Figure 1E) were detected in all membranes.

### Expression of MIF and Its Receptor CD74 in Epiretinal Fibrovascular Membranes From Patients With PDR

**Figure 2:**
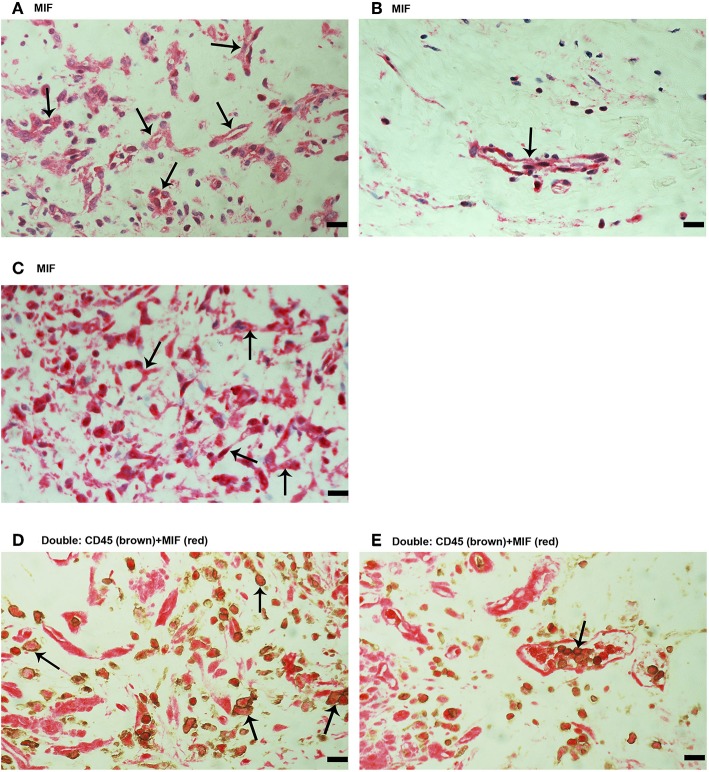
Characterization of macrophage migration inhibitory factor (MIF) expressing cells in proliferative diabetic retinopathy (PDR) epiretinal fibrovascular membranes. Immunohistochemical staining for MIF showing immunoreactivity in vascular endothelial cells (arrows) in a membrane from a patient with active angiogenesis **(A)** and in a membrane from a patient with involuted PDR **(B)**. Immunoreactivity for MIF was also detected in stromal spindle- shaped cells (arrows) **(C)**. Double immunohistochemistry for CD45 (brown) and MIF (red) demonstrated expression of MIF in stromal leukocytes (arrows) **(D)** and intravascular leukocytes (arrow) **(E)**. No counterstain to visualize the cell nuclei was applied **(D,E)**. (Scale bar, 10 μm).

Next, immunohistochemical analysis was used to reveal whether MIF, an inflammatory and angiogenic molecule associated with tumor pathology, is also expressed in the context of PDR. MIF immunoreactivity was observed in all membranes. Figure 2 shows representative images of membranes derived from patients with active (Figure 2A) or involuted (Figure 2B) disease. Immunoreactivity for MIF was noted in both endothelial cells lining blood vessels (Figures 2A,B) and stromal cells (Figure 2C). In the stroma, MIF expression was detected in spindle-shaped cells, as well as in CD45-expressing leukocytes. In serial sections, the distribution and morphology of spindle-shaped cells expressing MIF (Figure 2C) were similar to those of myofibroblasts expressing α-SMA (Figure 1E). Double immunostaining confirmed co-expression of CD45 and MIF in stromal and intravascular cells (Figures 2D,E).

**Figure 3:**
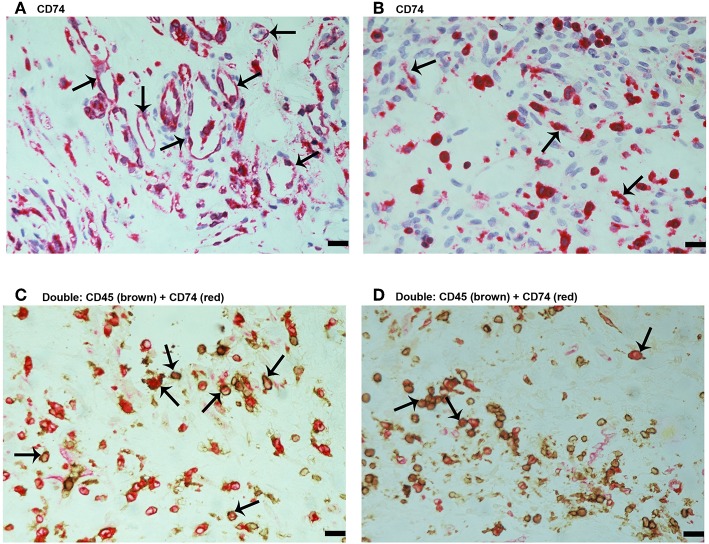
Characterization of CD74 expressing cells in proliferative diabetic retinopathy epiretinal fibrovascular membranes. CD74 Immunoreactivity was detected in vascular endothelial cells (arrows) **(A)** and in stromal spindle-shaped cells (arrows) **(B)**. Double immunohistochemistry for CD45 (brown) and CD74 (red) showed co-expression in stromal cells (arrows). No counterstain to visualize the cell nuclei was applied **(C,D)**. (Scale bar, 10 μm).

Since MIF activity requires binding to CD74 (15, 17), we also evaluated CD74 expression in epiretinal PDR membranes. CD74 immunoreactivity was detected in vascular endothelial cells (Figure 3A) and stromal cells (Figure 3B). CD74-expressing stromal cells were spindle-shaped cells (Figure 3B) resembling those being α-SMA positive in Figure 1E and leukocytes co-expressing CD45 (Figures 3C,D).

### Correlations Between Microvessel Density and the Expression of MIF and CD74 in Epiretinal Fibrovascular Membranes From Patients With PDR

Quantification of CD31-positive vessels in tumors is a standard method of measuring intra-tumoral microvessel density (MVD). Several studies reported that the level of MVD reflects the angiogenesis process in tumor tissues (22–24, 30, 31).

The mean number of blood vessels expressing CD31 was significantly higher in membranes from patients with active PDR than in membranes from patients with involuted PDR (Table 1) (Figures 1B,C). The numbers of blood vessels expressing MIF and stromal cells expressing MIF and CD74 were significantly higher in membranes from patients with active PDR than in membranes from patients with inactive PDR (Table 1) (Figures 2A,B).

**Table 1:** Mean number of immunoreactive blood vessels and stromal cells in epiretinal fibrovascular membranes in relation to the angiogenic activity of proliferative diabetic retinopathy (PDR).

**Variable**	**Active PDR** **(*n* = 9)** **Mean ± SD** **(Range)**	**Involuted PDR** **(*n* = 5)** **Mean ± SD** **(Range)**	***p*-value** **(Independent *t*-test)**
Blood vessels expressing CD31	110.8 ± 40.4 (75–175)	23.3 ± 10.4 (15-35)	<0.001^*^
Blood vessels expressing MIF	69.2 ± 10.2 (65–90)	42.9 ± 26.8 (15-84)	0.043^*^
Stromal cells expressing MIF	93.3 ± 31.3 (65–150)	39.0 ± 30.3 (6-80)	0.009^*^
Blood vessels expressing CD74	32.2 ± 19.5 (5-55)	21.8 ± 10.0 (11-35)	0.228
Stromal cells expressing CD74	150.6 ± 48.2 (80–215)	79.0 ± 51.3 (25-160)	0.023^*^

* *Statistically significant at 5% level of significance*.

*MIF, migration inhibitory factor*.

Significant positive correlations (Pearson correlation coefficients) were detected between the numbers of blood vessels expressing CD31 and the numbers of blood vessels expressing MIF (*r* = 0.56; *p* = 0.045) and stromal cells expressing MIF (*r* = 0.79; *p* = 0.001) and CD74 (*r* = 0.59; *p* = 0.045) (Table 2).

**Table 2:** Correlations (Pearson correlation coefficients) between microvessel density (MVD) and the numbers of immunoreactive blood vessels and stromal cells in proliferative diabetic retinopathy (PDR) epiretinal fibrovascular membranes.

	**Variable**	***r***	***p*-value**
MVD	Blood vessels expressing MIF	0.56	0.045^*^
	Stromal cells expressing MIF	0.79	0.001^*^
	Blood vessels expressing CD74	0.2	0.52
	Stromal cells expressing CD74	0.59	0.045^*^

* *Statistically significant at 5% level of significance*.

*MIF, migration inhibitory factor*.

### Immunohistochemical Analysis of PVR Epiretinal Fibrocellular Membranes

**Figure 4:**
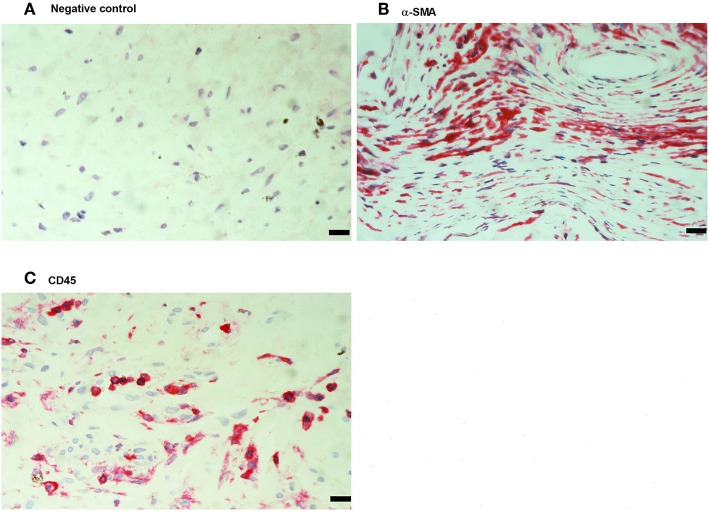
Detection of myofibroblasts and leukocytes in proliferative vitreoretinopathy (PVR) epiretinal fibrocellular membranes. Negative control slide showing no labeling **(A)**. Immunohistochemical staining for α-smooth muscle actin (α-SMA) showing immunoreactivity in spindle-shaped myofibroblasts **(B)**. Immunohistochemical staining for CD45 showing numerous leukocytes in the stroma **(C)**. (Scale bar, 10 μm).

**Figure 5:**
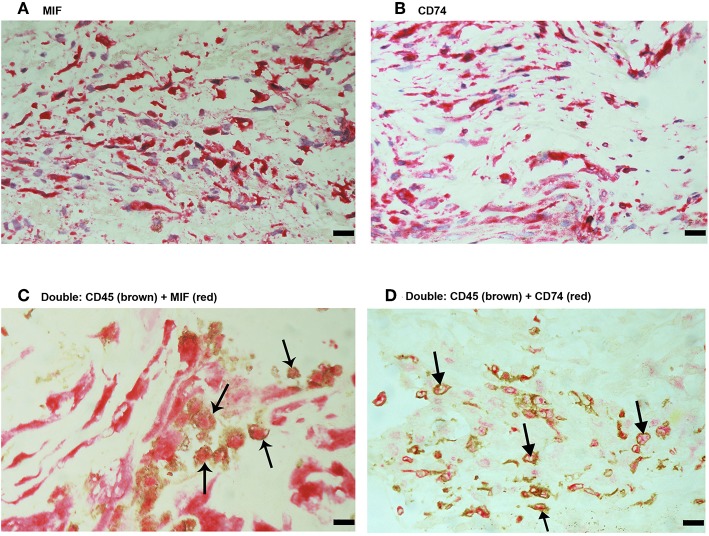
Characterization of macrophage migration inhibitory factor-(MIF-) and CD74-expressing cells in proliferative vitreoretinopathy (PVR) epiretinal fibrocellular membranes. Immunohistochemical staining for MIF **(A)** and CD74 **(B)** showing immunoreactivity in spindle-shaped myofibroblasts. Double Immunohistochemistry for CD45 (brown) and MIF (red) **(C)** or CD74 (red) **(D)** demonstrated co-expression in leukocytes. No counterstain to visualize the cell nuclei was applied (arrows) **(C,D)**. (Scale bar, 10 μm).

No immunoreactivity was detected in negative control slides (Figure 4A). All PVR membranes contained spindle-shaped α-SMA^+^ myofibroblasts (Figure 4B) and CD45^+^ leukocytes (Figure 4C). Immunostainings for MIF (Figure 5A) and CD74 (Figure 5B) revealed spindle-shaped cells expressing cytoplasmic immunoreactivity, which were in distribution and morphology similar to the α-SMA^+^ myofibroblasts (Figure 4B) in serial sections. In addition, double-labeling experiments showed that cells expressing MIF (Figure 5C) and CD74 (Figure 5D) co-expressed CD45.

### Levels of MIF, VEGF, and sICAM-1 in Vitreous Samples

In addition, we used ELISA to compare MIF levels in vitreous samples from 36 patients with PDR to those of 20 nondiabetic controls. MIF was detected in 17 of 20 (85%) vitreous samples from nondiabetic controls, and in all vitreous samples from patients with PDR. In nondiabetic controls, we found a median [IQR] level of 3.2 [1.7–4.6] ng/ml. In contrast, the median [IQR] concentration in patients with PDR reached 15.4 [10.0–20.0] ng/ml, a concentration approximately 5-fold higher than that recorded in nondiabetic controls (*p* < 0.001; Mann-Whitney test) (Table 3).

**Table 3:** Comparisons of migration inhibitory factor (MIF), vascular endothelial growth factor (VEGF) and soluble intercellular adhesion molecule-1 (sICAM-1) in vitreous samples from patients with proliferative diabetic retinopathy (PDR) and nondiabetic patients with rhegmatogenous retinal detachment (RD).

	**PDR** **(*n* = 36)** **Median (IQR)**	**RD** **(*n* = 20)** **Median (IQR)**	***p*-value** **(Mann-Whitney test)**
MIF (ng/ml)	15.4 (10.0–20.1)	3.2 (1.7–4.6)	<0.001^*^
VEGF (pg/ml)	356.0 (151.0–712.3)	1.5 (0.0–18.3)	<0.001^*^
sICAM-1 (ng/ml)	17.2 (6.2–24.0)	4.1 (1.0–9.2)	<0.001^*^

* *Statistically significant at 5% level of significance*.

*IQR, interquartile range (Q1–Q3)*.

The angiogenic biomarker VEGF was detected in 9 of 20 (45%) vitreous samples from nondiabetic controls and in 33 of 36 (91.6%) vitreous samples from PDR patients. The proinflammatory biomarker sICAM-1 was detected in 18 of 20 (90%) vitreous samples from nondiabetic controls and in all vitreous samples from patients with PDR. The levels of VEGF and sICAM-1 were significantly higher in PDR (*p* < 0.001 for both comparisons; Mann-Whitney test) (Table 3).

**Figure 6:**
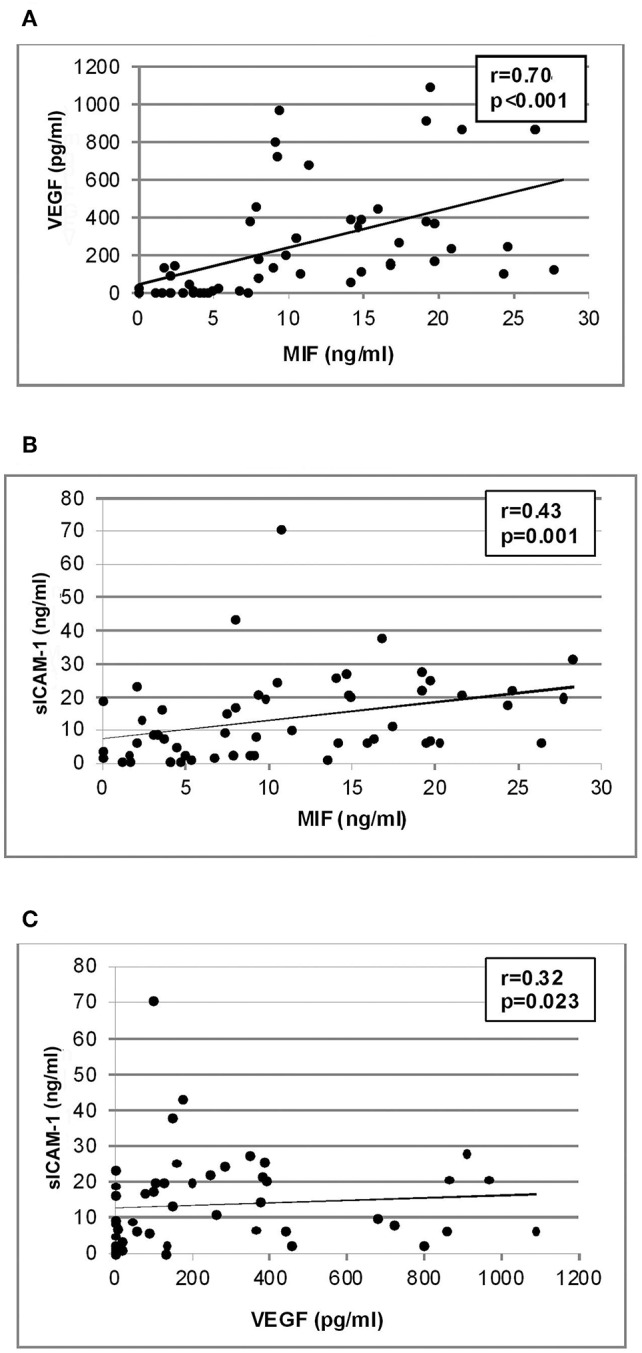
Correlation analyses to reveal interactions between vitreous fluid levels of migration inhibitory factor (MIF), vascular endothelial growth factor (VEGF) and soluble intercellular adhesion molecule-1 (sICAM-1). Significant positive correlations between vitreous fluid levels of MIF and levels of VEGF **(A)** and sICAM-1 **(B)** and between vitreous fluid levels of VEGF and levels of sICAM-1 **(C)** were found.

Significant positive correlations (Spearman's correlation coefficient) were found between vitreous fluid levels of MIF and levels of VEGF (*r* = 0.70; *p* < 0.001) and sICAM-1 (*r* = 0.43; *p* = 0.001). In addition, a significant positive correlation was observed between vitreous fluid levels of VEGF and the levels of sICAM-1 (*r* = 0.32; *p* = 0.023). Although the correlations between MIF and sICAM-1 and between VEGF and sICAM-1 were weak, yet they were statistically significant (Figure 6).

### Relationship Between ELISA Levels of MIF in Vitreous Samples and Angiogenic Activity of PDR

Comparisons of MIF levels among PDR patients with active neovascularization (*n* = 20), PDR patients with involuted (quiescent) neovascularization (*n* = 16), and nondiabetic controls (*n* = 20) was conducted with the Kruskal-Wallis test. The levels differed significantly between the 3 groups (*p* < 0.001). Pairwise comparisons (Mann-Whitney test) demonstrated that the median [IQR] MIF level was significantly higher in active PDR (19.6 [15.4–24.5] ng/ml) than in involuted PDR (9.6 [8.3–14.7] ng/ml) (*p* < 0.001) and controls (3.2 [1.7–4.6] ng/ml) (*p* < 0.001). Furthermore, the median MIF level in involuted PDR was significantly higher than in controls (*p* < 0.001).

### Detection of sCD74 in Vitreous Samples

**Figure 7:**
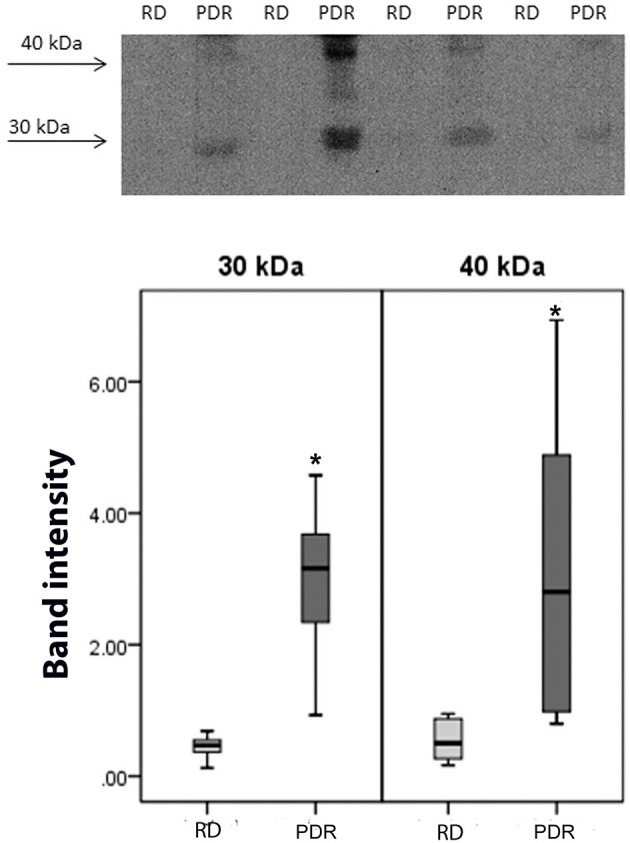
Detection of soluble (s) CD74 in vitreous fluid. The expression of sCD74 in vitreous samples from patients with proliferative diabetic retinopathy (PDR; *n* = 8) and from nondiabetic patients with rhegmatogenous retinal detachment (RD; *n* = 8) was determined by Western blot analysis. A representative set of samples is shown. Densitometric analysis of the bands demonstrated a significant increase in both the sCD74 isoforms with molecular weights of around 30 and 40 kDa in samples from PDR patients compared to samples from nondiabetic control patients. Results are expressed as median (interquartile range) (*p < 0.05).

Using Western blot analysis, we demonstrated the presence of sCD74 in vitreous samples from patients with PDR. In agreement with previous studies (39, 40), sCD74 protein was detected as two protein bands with molecular weights of around 30 and 40 kDa. Densitometric analysis of the bands demonstrated a significant increase in both the 30 kDa band (*p* = 0.002; Mann-Whitney test) and the 40 kDa band (*p* = 0.006; Mann-Whitney test) intensities in samples from PDR patients (*n* = 8) compared to samples from nondiabetic control patients (*n* = 8) (Figure 7).

### MIF Induces Upregulation of VEGF and Phospho-ERK1/2 in Retinal Müller Cells

**Figure 8:**
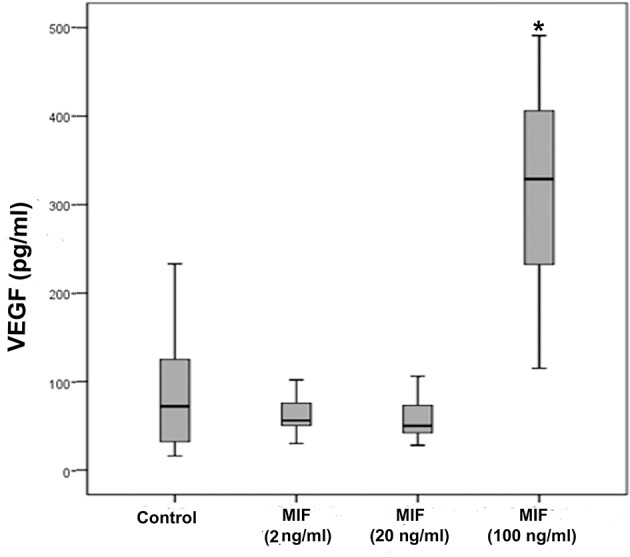
Macrophage migration inhibitory factor (MIF) induces vascular endothelial growth factor (VEGF) expression in Müller cells. Müller cells were left untreated or treated with MIF (2, 20 or 100 ng/ml) for 24 h. Levels of VEGF were quantified in the culture media by ELISA. Results are expressed as median (interquartile range) from three different experiments. In each experiment, every experimental condition was included 8 times. (*p < 0.05 compared to the values obtained from untreated cells).

**Figure 9:**
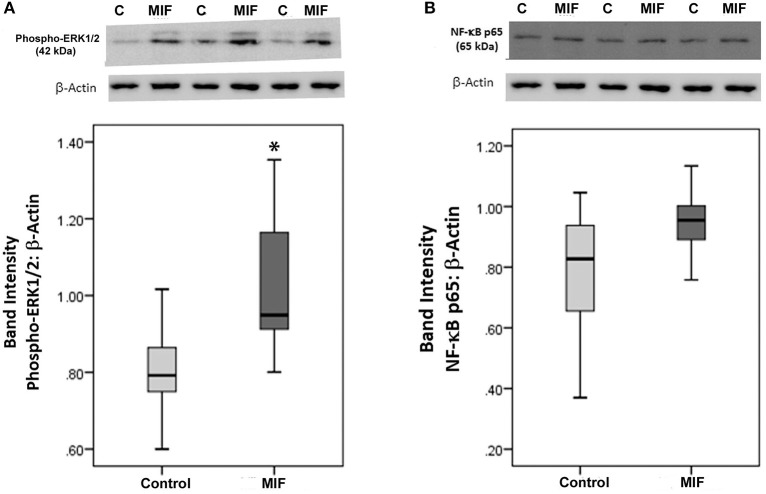
Macrophage migration inhibitory factor (MIF) activates the ERK1/2 pathway in Müller cells. Müller cells were left untreated or treated with MIF (100 ng/ml) for 24 h. Protein expression of phospho-ERK1/2 **(A)** and p65 subunit of NF-κB **(B)** in the cell lysates was determined by Western blot analysis. After transfer of the proteins to the nitrocellulose membrane, the membrane was cut horizontally and the lower and upper part were stained with anti-phospo-ERK1/2 and anti-NFκB, respectively. Afterwards, the lower part of the membrane was stripped and reprobed with anti-β-actin. Therefore, phospho-ERK1/2 **(A)** and p65 subunit of NF-κB **(B)** expression were normalized to the same loading control. Results are expressed as median (interquartile range) from three different experiments. In each experiment, every experimental condition was tested in quadruplicate (*p < 0.05 compared with the values obtained from untreated cells).

As we observed a positive correlation between the vitreous fluid levels of MIF and VEGF, we performed short-term induction experiments on Müller cells with MIF as an inducer of VEGF production. At 100 ng/ml, MIF significantly enhanced the levels of VEGF in the culture medium (Figure 8). However, 2 and 20 ng/ml MIF did not affect the expression of VEGF as compared to untreated control (Figure 8). Western blot analysis demonstrated that treatment of Müller cells with MIF (100 ng/ml) induced significant upregulation of the protein levels of phospho-ERK1/2 (Figure 9A). In contrast, expression of the p65 subunit of NF-κB was not significantly altered (*p* = 0.057; Mann-Whitney test) (Figure 9B).

### The Hypoxia Mimetic Agent CoCl_2_ Induces Upregulation of MIF and VEGF in Retinal Müller Cells

**Figure 10:**
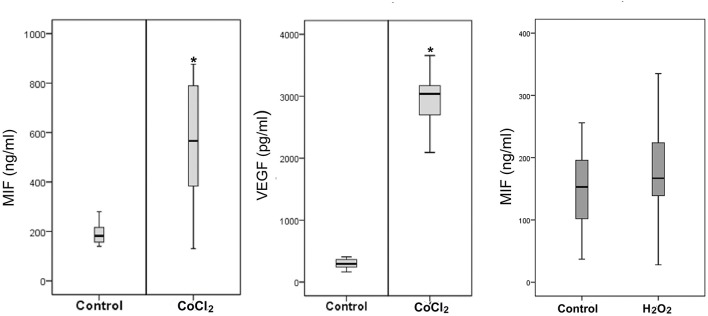
Hypoxia induces production of macrophage migration inhibitory factor (MIF) and vascular endothelial growth factor (VEGF) by Müller cells. Müller cells were left untreated or treated with cobalt chloride (CoCl_2_) (300 μM) or hydrogen peroxide (H_2_O_2_) (100 μM) for 24 h. Levels of MIF and VEGF were quantified in the culture media by ELISA. Results are expressed as median (interquartile range) from three different experiments. In each experiment, every experimental condition was included 8 times (*p < 0.05 compared to the values obtained from untreated cells).

Retinal Müller cells are not only a major source of VEGF (41), these cells also produced enhanced amounts of MIF in addition to VEGF after treatment with 300 μM of CoCl_2_ (Figure 10). However, oxidative stress, mimicked by addition of 100 μM of H_2_O_2_ to the Müller cell cultures did not affect the expression of MIF as compared to untreated control (Figure 10).

### MIF Induces Migration and Proliferation of Human Retinal Microvascular Endothelial Cells

**Figure 11:**
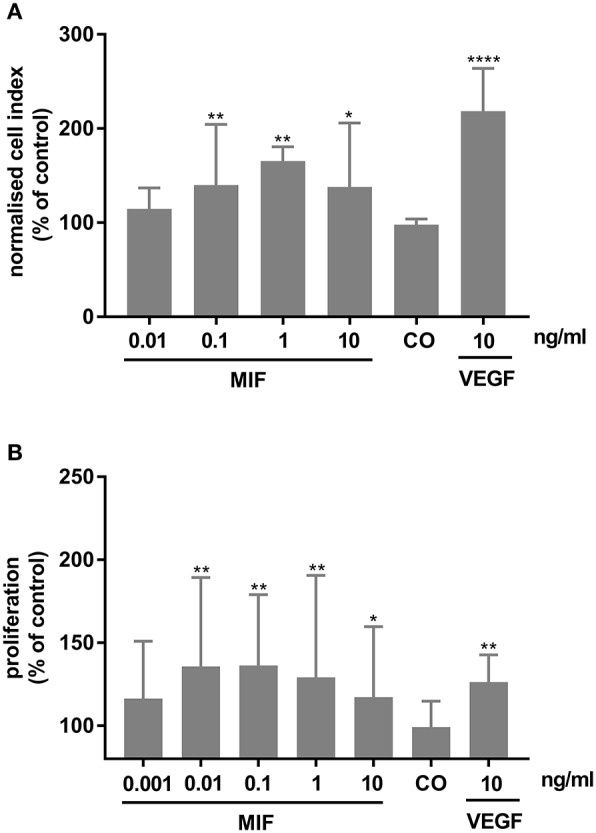
Macrophage migration inhibitory factor (MIF) stimulates migration and proliferation of human retinal microvascular endothelial cells (HRMECs). **(A)** HRMECs were stimulated with dilution medium alone (CO), MIF (0.01, 0.1, 1, 10 ng/ml) or VEGF (10 ng/ml). Electrical impedance was measured every minute for 15 h using the xCELLigence RTCA-DP system and automatically converted to cell indices by the xCELLigence software. The results shown are derived from the time point where the maximal cell index was reached (about 12 h after initiation). The median (interquartile range) percentage of migration compared to control is shown (*n* ≥ 3). **(B)** The number of HRMECs was quantified 48 h after addition of dilution medium, VEGF (10 ng/ml) or MIF (0.001 to 10 ng/ml). Results are expressed relative to control (untreated cells). The relative proliferation is shown as median (interquartile range) (*n* = 4 in triplicate). (****p < 0.0001; **p < 0.01; *p < 0.05 compared to unstimulated cells).

As MIF has been described to be involved in pathological neovascularization of tumors, we tested in the following experiments whether HRMECs are also responsive to MIF. When added to the lower compartment of a chemotaxis chamber, MIF rather potently induced migration of the HRMECs. Indeed, the minimal effective dose required to trigger a significant chemotactic response was only 0.1 ng/ml. VEGF, however, was a more efficient chemoattractant (Figure 11A). Furthermore, MIF also induced proliferation of HRMECs. When 0.01 ng/ml or higher concentrations of MIF were added to the endothelial cell cultures, proliferation increased on average to 60% above background (Figure 11B).

### *In vivo* Effect of Intravitreal Administration of MIF

**Figure 12:**
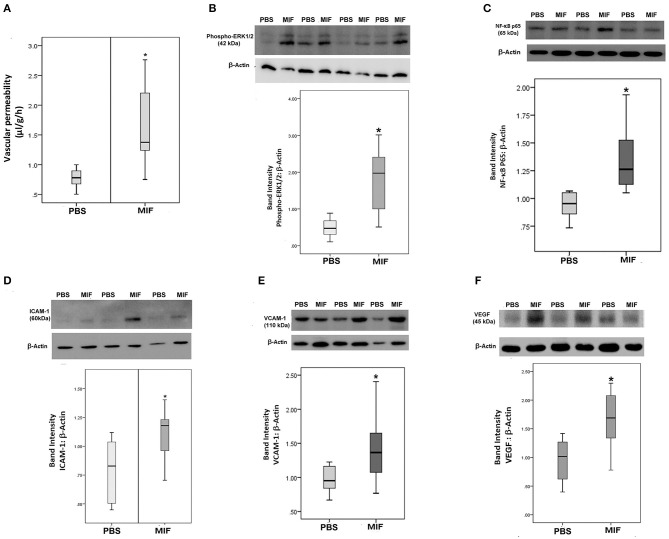
Macrophage migration inhibitory factor (MIF) induces blood-retinal barrier (BRB) breakdown and *in vivo* activation of inflammatory pathway. MIF (*n* = 12) was injected intravitreally at the dose of 5 ng in 5 μl in one eye and the same volume of phosphate buffer saline (PBS; *n* = 11) was injected in the contralateral eye of normal rats. BRB breakdown was quantified with the FITC-conjugated dextran technique **(A)**. As demonstrated by Western blot analysis of rat retinas, intravitreal administration of MIF induced a significant upregulation of the expression of phospho-ERK1/2 **(B)**, p65 subunit of NF-κB **(C)**, intercellular adhesion molecule-1 (ICAM-1) **(D)**, vascular cell adhesion molecule-1 (VCAM-1) **(E)** and vascular endothelial growth factor (VEGF) **(F)** compared with intravitreal administration of PBS. Results are expressed as median (interquartile range) (*p < 0.05 compared to the values obtained from PBS-injected eyes).

Finally, 5 ng of MIF was injected in the eyes of normal rats. Fluorescein isothiocyanate-conjugated dextran was used to determine the subsequent change in vascular permeability. Figure 12A shows that intravitreal administration of MIF (*n* = 12) significantly increased retinal vascular permeability by about two-fold compared with vehicle (PBS)-injected eyes (*n* = 11). Furthermore, MIF induced significant upregulation of the protein levels of phospho-ERK1/2 (Figure 12B), the p65 subunit of NF-κB (Figure 12C), ICAM-1 (Figure 12D), VCAM-1 (Figure 12E), and VEGF (Figure 12F) in the retinas, compared to the values obtained from the contralateral eyes that received PBS alone.

## Discussion

In the current study, we detected for the first time co-expression of MIF and its receptor CD74 in endothelial cells, leukocytes and myofibroblasts in epiretinal fibrovascular membranes from patients with PDR. In addition, there were significant positive correlations between the levels of MIF and CD74 expression and the angiogenic activity in PDR epiretinal fibrovascular membranes. Our immunohistochemical data are in line with those of previous studies that demonstrated MIF and CD74 expression by tumor-associated fibroblasts, leukocytes and endothelial cells (27). Several studies proposed that the MIF/CD74 signaling pathway is an important regulator in pathological tumor-associated angiogenesis (21–23, 27) and showed that MIF expression levels correlated with tumor angiogenesis (30, 31). In animal models of cancer, it was demonstrated that MIF had the potential to promote tumor growth and tumor-associated angiogenesis and that anti-MIF antibodies suppressed angiogenesis in these models (33). Several reports demonstrated that MIF directly and potently induces angiogenesis in multiple *in vitro* and *in vivo* models (24–26). In the present study, we demonstrated that MIF induced HRMEC migration and proliferation, early key steps during angiogenesis. These findings implied that MIF and CD74 might play roles as a ligand-receptor complex in PDR angiogenesis. Previously, it has been shown that MIF expression was also upregulated in an animal model of corneal neovascularization and that MIF-deficient mice had less neovascularization (42). In a mouse model of oxygen-induced ischemic retinal neovascularization, MIF deficiency reduced pathological preretinal angiogenesis and the expression of proinflammatory and proangiogenic factors (43).

In this study, we also demonstrated that MIF and sCD74 were significantly upregulated in the vitreous fluid from patients with PDR, and that vitreous MIF levels were significantly higher in PDR eyes with active neovascularization compared with eyes with quiescent disease. Our findings are in agreement with a previous study that demonstrated upregulated expression of MIF in the vitreous fluid from patients with PDR (44). Our analysis showed significant positive correlations between the vitreous fluid levels of MIF and those of the inflammatory biomarker sICAM-1 and the angiogenic biomarker VEGF. In addition, we demonstrated that intravitreal injection of MIF in normal rats induced significant upregulation of ICAM-1 and VCAM-1 in the retina. Our findings are consistent with previous reports documenting the role of MIF in upregulating ICAM-1 and VCAM-1 in different types of cells (45, 46). In an animal model of retinal detachment, MIF was identified by a proteomics screen to be the most important cytokine upregulated in retinal detachment. Administration of a MIF inhibitor blocked pathological damage responses by protecting photoreceptors and reducing gliosis (47).

Among the proangiogenic factors, VEGF is considered as the most potent one with a pivotal role in PDR (10, 11). To corroborate the findings at the cellular level, stimulation with MIF caused upregulation of VEGF in Müller cells. Müller cells are considered to contribute to pathological retinal neovascularization by being the principle VEGF-producing cell type (41). In addition, Matsuda et al. demonstrated expression of MIF in rat Müller cells (48). To our knowledge, the present study is the first to report the capability of MIF to target Müller cells and to induce upregulation of phospho-ERK1/2 and the synthesis and secretion of VEGF. Additionally, intravitreal injection of MIF induced a significant upregulation of VEGF, phospho-ERK1/2 and the p65 subunit of NF-κB in the retina of rats. This is in line with previous studies documenting the capacity of MIF to induce VEGF and phosphorylation of ERK1/2 in tumor cells (23, 28–30). These findings suggest that one possible mechanism of MIF-induced angiogenesis in PDR is related to the upregulation of VEGF. In addition, upregulation of VEGF is a major contributor to BRB breakdown in diabetes (49–51). Our findings also suggest that MIF-induced BRB breakdown might be related to upregulation of VEGF. Furthermore, we showed that stimulation with the hypoxia mimetic agent CoC1_2_ promoted VEGF and MIF expressions in Müller cells. Similarly, previous studies demonstrated that MIF was induced by hypoxia in cancer cells (30, 52, 53).

In addition to its well characterized role in inflammation and angiogenesis, MIF is upregulated in fibrotic disorders, such as idiopathic pulmonary fibrosis (54, 55) and systemic sclerosis (56). Additionally, MIF expression increased during the wound healing of rat skin injured by excision and anti-MIF antibodies induced a delay in wound healing (57). In the present study, immunohistochemical analysis demonstrated MIF and CD74 localization in myofibroblasts, the key cellular mediator of fibrosis (58), in epiretinal membranes from patients with PDR and PVR. Similarly, dermal fibroblasts from skin wound lesions (57) and systemic sclerosis (56) produced higher amounts of MIF than normal dermal fibroblasts. It was demonstrated i*n vitro* that transient exposure of fibroblasts to MIF induced fibroblast activation and promoted fibroblast proliferation, migration and replenishment of cell monolayers after scratching (59). In a recent study, an interesting genetic association of MIF with epiretinal membrane formation was found, suggesting a potential contribution of MIF to formation of the membranes (60).

In conclusion, the proinflammatory and proangiogenic cytokine MIF and its receptor CD74 are upregulated in the intraocular microenvironment of patients with PDR, particularly in patients with active angiogenesis. Additionally, intravitreal administration of MIF significantly increased retinal vascular permeability in rats. MIF stimulation of Müller cells induced increased secretion of VEGF and CoC1_2_ induced the production of MIF from Müller cells *in vitro*. Stimulation of endothelial cells isolated from human retinas with MIF induced migration and proliferation, confirming its reported angiogenic effects. Therefore, the MIF/CD74 signaling pathway might play an important role in PDR angiogenesis and progression and could become a primary therapeutic target for improving the vascular function in patients with PDR.

## Data Availability Statement

The datasets generated for this study are available on request to the corresponding author.

## Ethics Statement

The study was conducted according to the tenets of the Declaration of Helsinki. Before undergoing vitrectomy, all patients signed a preoperative informed written consent and approved the use of the excised epiretinal membranes and aspirated vitreous fluid for further analysis and clinical research. The Research Centre and Institutional Review Board of the College of Medicine, King Saud University approved the study design and protocol.

## Author Contributions

AMA designed the manuscript, supplied funding, interpreted the data, and wrote the manuscript. AA, MS, EA, and AD performed experiments and interpreted the data. PG analyzed the data. GD designed, supervised, and interpreted IHC stainings. JV designed experiments. GO provided funding and designed experiments. SS provided funding, designed experiments, interpreted the data, and wrote the manuscript. All authors read and approved the final manuscript.

### Conflict of Interest

The authors declare that the research was conducted in the absence of any commercial or financial relationships that could be construed as a potential conflict of interest.
